# How to optimize the design and implementation of risk prediction tools: focus group with patients with IgA nephropathy

**DOI:** 10.1186/s12911-020-01253-4

**Published:** 2020-09-16

**Authors:** Anna R. Gagliardi, Heather N. Reich, Daniel C. Cattran, Sean J. Barbour

**Affiliations:** 1 University Health Network, Toronto, Canada; 2 University of British Columbia, Vancouver, Canada

**Keywords:** Chronic kidney disease, IgA nephropathy, Patient-centred care, Patient-provider communication, Risk prediction calculator, User-centred design, Qualitative research

## Abstract

**Background:**

IgA nephropathy (IgAN) is a common type of chronic immune-mediated kidney disease with variable risk of progression to end-stage kidney disease. Risk stratification helps clinicians weight the potential risks and benefits of immunosuppressive therapy for individual patients, and can inform patient-centred communication. No prior research examined barriers of risk predication tools (RPT) specific to IgAN. The purpose of this study was to explore determinants (facilitators, barriers) of RPT use from the patient perspective.

**Methods:**

We conducted a single focus group with English-speaking adults aged 18 or older with biopsy-proven IgAN. We asked about how they would use an IgAN RPT, and how to improve its design and implementation. We analyzed the transcript using constant comparison to inductively derive themes, and complied with qualitative research reporting criteria.

**Results:**

The 5 participants were Caucasian men who varied in age from 35 to 55. The glomerular filtration rate ranged from 29 to 71 mL/min/1.73m^2^, and proteinuria ranged from 0.36 to 1.41 g/d. Participants identified both benefits and harms of the risk score. They said physicians should first ask patients for permission to use it. To make it more useful, participants offered suggestions to enhance RTP design: visual display, information on how to interpret the risk score, risk categories, health implications, modifiable risk factors, multiple scenarios, and comparison with similar patients. They offered additional suggestions to enhance RPT implementation: it should not replace patient-provider discussion, it should be accompanied by self-management education so that patients can take an active role in their health. Participants appreciated information from members of the multidisciplinary team in addition to physicians. Participants also said that physicians should monitor patient emotions or concerns on an ongoing basis.

**Conclusions:**

Patients with IgAN identified numerous ways to enhance the design and use of an RPT. Others could use this information to design and implement RPTs for patients with other conditions, but should employ user-centred design to develop RPTs that address patient preferences.

## Background

Glomerulonephritis is a general term for autoimmune-based kidney diseases. The most common type of glomerulonephritis worldwide is IgA nephropathy (IgAN), with patients diagnosed across the age spectrum, and presenting at a mean age of 37 years [1]. Disease progression of IgAN to end-stage kidney disease requiring dialysis is a devastating complication for such a young patient population. A significant challenge in IgAN is that individual risk of progression to end-stage kidney disease is highly variable, ranging between < 10 and > 60% at 10 years [2]. Although effective immunosuppressive therapies are available, they are highly toxic with severe side effects [3]. As such, guidelines recommend each patient be risk stratified so that treatment can be targeted to high-risk patients most likely to benefit [4]. Although clinical risk factors for end-stage kidney disease are well established and readily available, they are very poor at predicting risk of disease progression, and relying on them to guide treatment decisions results in significant patient harm through inappropriate over or under treatment [5–7].

To more accurately predict disease progression in IgAN and support better decision-making, leading to improved care delivery and optimal patient outcomes, our research group developed the International IgAN Prediction Tool [8]. This initiative represents a large international collaboration of investigators from China, Japan, Europe, North and South America, collectively referred to as the International IgAN Network, whose goal was to merge research databases in order to provide the infrastructure necessary to support the development and validation of the Prediction Tool. This model uses predictor variables readily available in clinical practice at the time of IgAN diagnosis, can accurately predict the risk of a 50% decline in kidney function or end-stage kidney disease, and could be employed as the basis for patient-provider communication and decision-making [8].

Risk prediction tools (RPTs) are one way to support patient-centred care (PCC), which is widely advocated because it enhances patient-important and clinical outcomes such as increased knowledge, treatment adherence, and quality of life; and reduced anxiety, readmission rates and mortality [9–11]. PCC is defined as partnership among practitioners, patients and their families to ensure that providers and systems deliver care that is attentive to the needs, values and preferences of patients [12]. By clarifying details about prognosis, RPTs can foster strong patient-provider communication and care tailored to patient preferences. While the number of available RPTs has considerably increased over the past decade, particularly for diabetes and cardiovascular disease, research shows that they are not used in clinical practice [13–16]. As a result, others have argued for more research on factors that influence the adoption and use of RPTs, and how to implement them in order to maximize benefits and minimize potential harms such as over-medicalization, false assurances and anxiety [13, 14, 17, 18]. Barriers of RPT use are present at the physician (threat to physician decision-making authority, evidence not provided on determinants of predicted risk, perceived importance of clinical issue, practicality of integrating tools into practice) and patient (understanding results, concerns about results) levels [19–21].

The IgAN Prediction Tool has been integrated into the Calculate app by QxMD (www.qxmd.com), which is a mobile app calculator that targets physicians as the knowledge users. Because of the complexity of predictor variable input and the format of risk prediction output, the mobile app is likely not suitable for direct patient use. To address this limitation, the International IgAN Network plans to develop a patient-oriented web-based education tool that includes the Prediction Tool models presented in way specifically designed to meet patient needs, inform them of their anticipated prognosis, and empower them to engage in informed discussions and shared decision making with their care team.

The existing literature suggests that that RPTs require physicians to purposefully integrate their clinical experience with probabilistic data, weigh the risks and benefits for a given patient, reach a decision, and translate all of that for patients to support patient engagement in decision-making, which is complex and challenging [19]. However, these barriers were revealed in studies of RPTs for post-operative nausea [19], diabetes [20], and physical therapy [21], none of which are associated with such clinically profound implications as IgAN. Given that there is no research on factors that influence the adoption and use of RPTs by patients with IgAN, and factors influencing patient use of RPTs may differ by disease, there is no evidence to inform the development of the patient education tool by the International IgAN Network. As such, the purpose of this study was to explore determinants (barriers/facilitators) of the use of the IgAN Prediction Tool (henceforth, IgAN RPT) among patients with IgAN. This knowledge would offer insight on how to design and implement the IgAN RPT, and will inform the design of a patient-oriented education tool that in future research can be pilot tested and trialed for use in clinical care.

## Methods

### Approach

This research comprised the first step in developing a patient-oriented education tool. The overall approach was based on two widely-used, complementary approaches to develop and evaluate such interventions: Framework for the Design and Evaluation of Complex Interventions [22], and user-centred design, which involves end-users in all development steps [23]. Step one, concept generation, refers to generating evidence to inform intervention design, often based on qualitative research with a small number of participants. Subsequent steps, including intervention development, pilot-testing and more definitive trial-based evaluation, then include a broader range of participants. In this case, given the paucity of prior research on the views of IgAN patients about risk prediction, we employed a qualitative design to thoroughly explore patient experiences and suggestions related to the value of this information to patients [24]. More specifically, a basic qualitative descriptive approach was used [25]. Unlike other qualitative approaches that employ or generate theory, this technique elicits straightforward descriptions of lived experiences. We conducted an in-person focus group rather than individual interviews because interaction amongst participants encourages rich, synergistic discussion about common and differing views [26]. We complied with the 32-item Consolidated Criteria for Reporting Qualitative Research [27]. We further ensured rigour through independent coding and review of data by the research team, and assessment of discrepant experiences and suggestions [28]. The research team was composed of nephrologists, and a PhD-trained health services research with expertise in implementation science and qualitative research (ARG). The University Health Network Research Ethics Board approved the study. We informed all participants about the study’s purpose and provided written informed consent prior to the focus group. There was no prior relationship between the interviewer and participants.

### Sampling and recruitment

We used convenience sampling to recruit English-speaking adult persons (18+ years) diagnosed with biopsy-proven IgAN, and with an estimated GFR (eGFR) > =20 ml/min/1.73m^2^ so that the disease was not considered so advanced that risk prediction was no longer relevant. We excluded patients with both an eGFR> 90 ml/min/.173m^2^ and proteinuria< 0.5 g/day because these patients have minimal disease activity and an extremely favourable long-term kidney prognosis such that risk prediction is not relevant. We identified and recruited eligible persons at the University Health Network Glomerular Disease Clinic by a clinic nurse working with two nephrologists (HR, DC). The clinic nurse obtained written, signed consent and then provided contact details and demographic characteristics for consenting persons to ARG. Thus, physicians on our research team were unaware of who from among their practice took part in focus groups. There was no prior relationship between ARG and focus group participants. We aimed to recruit 6 to 8 persons, a common size for focus groups [26]. We aimed to recruit persons who varied in non-mutually exclusive fashion for demographic characteristics that could influence their views and experiences such as age, gender, ethnicity, and measures of disease severity based on eGFR and proteinuria. Recruitment was launched on June 10, 2018 and concluded on July 10, 2019.

### Data collection

ARG conducted a single one-hour focus group on August 13, 2019 in a meeting room at the hospital where participants received care to provide a familiar, accessible environment. First ARG explained the purpose of the study, defined a risk calculator, and showed an online version of the IgAN RPT and its output (https://qxmd.com/calculate/calculator_499/international-igan-prediction-tool). ARG then asked four questions: *How would you use information about whether you do or do not have a future risk of worsening kidney function? How could the risk calculator help you discuss your health with your doctor? How could the format and output of the risk calculator be improved so that it is more useful?* and *In addition to the risk calculator, is there anything else that would help patients discuss the risk calculator results with their nephrologist?* The focus group was audio-recorded and transcribed by a professional transcriptionist.

### Data analysis

ARG and SB derived themes inductively from the data using constant comparison [24]. Each independently read the transcript to identify and code all themes, discussed and agreed upon themes, and created a codebook of themes and corresponding quotes in Microsoft Word. The research team reviewed themes and exemplar quotes to assist with interpretation of the findings.

## Results

### Participants

In total, 5 persons participated in the focus group. They varied by age (range 35 to 55) and health status (Table 1).

**Table 1:** Focus group participant characteristics

Gender	Age	Race	eGFR (mL/min/1.73m^2^)	Proteinuria (g/d)	Kidney biopsy	Ever treated with immuno-suppression	Currently on immuno-suppression	On renin-angiotensin system blocker
Male	55	Caucasian	29	0.40	Sept 2009	Yes	Yes	Yes
Male	41	Caucasian	60	0.63	Apr 1998	No	No	Yes
Male	47	Caucasian	55	0.36	Oct 2014	Yes	Yes	Yes
Male	35	Caucasian	71	1.41	Sept 2011	Yes	No	Yes
Male	54	Caucasian	56	0.65	Oct 2016	Yes	No	Yes

### Themes by PCC domain

Themes and exemplar quotes by PCC domain are included in Table 2 and discussed here.

**Table 2:** Themes and exemplar quotes

Themes	Sub-themes and exemplar quotes
Value of risk score	Advantage over current self-monitoring • The information isn’t of use to me, speaking personally. I get the copies of the lab results. I track them on my cell to see how progression is. I think they’re moving away from the old model whereby you know the doctor has the information, tells the patient what to do • did we need the risk calculator to get us on track and just have more self awareness? No, I think we just had the right communication with our physician and the right information on how to interpret results from the lab • for me the number is a very small part of the story and I want to know what I can control and what’s potentially controllable because that’s what I can act on. If I’m just given a number, I mean the number itself doesn’t really do anything to help me practically. It just gives you an idea of the statistical likelihood of something happening Any information is useful • I think it’s useful for a patient to have that information as well • More information is better. I like to know as much as possible. • I don’t see any disadvantages; I see many positives to sharing this information with patients
Benefits of risk score	Understand health status • it just gives you a sense of direction or piece of mind I guess, knowing what your numbers are. When you get diagnosed, you want to know more, you want to know, ‘Okay, what the hell, what am I doing? Where am I going? What’s my direction here?’ So a little bit more of that is always a positive • it reflects on what the status is and the prognosis; it’s a fairly measurable quantitative assessment of where you are right now Periodic use could show changes or improvements • my early diagnosis would have had a very high score of potential failure within a short period of time; treatment began and we levelled off. And I would suggest that if we were to repeat that, my score would now be lower than it was. So I think knowing at the beginning and then repeating the, to show we are making progress or we are at least managing the condition would be a great indicator for me. It would show the efficacy of the treatment that I’m receiving • What is the frequency that we do this calculator? If I do it every week am I gonna benefit? And then plotting the outcome of this over a five year period where I’ve done it every three months or every six months and now I can see the curve changing to flatter or zero. If it changes to zero or if it goes negative, and I growing new kidneys? So anything like that to give me a clue? Influence life choices • certainly would make different life choices based on any percentage chance if I thought of having a significant risk of dialysis for … even a transplants … accelerate travel choices, the lifestyle choices, job choices and frankly would be quite a significant set of changes based on that information • So renal failure within 10-years, I’d probably die of something else. Renal failure within 15 or 16 or 18 months, I’m gonna maybe make some choices, changes Basis for patient-provider discussions • These are the kinds of conversations that the physician and the patient need to have in the early stages of diagnosis because an education, the education curve is steep to try and understand renal diseases, not a simple thing and to try and get your head around all the metrics that we all track is a considerably complicated activity to learn to manage. So in the initial conversations, keep it simple, and let’s gradually add complexity over time to help me better adapt to the onslaught of information that you get on the first day of diagnosis. • it is definitely overwhelming, the number of metrics you have to understand and learn about. It maybe a good tool to just summarize because it is a really overwhelming and as soon as you get that diagnosis, it would be simple enough to have a number • I was very fortunate to have somebody, like a team, the physician and the research scientist or technician, I’m not sure, help me interpret these things. So it wasn’t always a physician Prompts discussion when physicians not forthcoming with information • I would imagine that there is some unevenness throughout the province; this could be a way of going about standardizing it, simplifying it as necessary to deal with the amount of information. But also to make the information as available • if it acts as a conversation starter, or someone asking questions, then that’s good. I guess a patient asking questions is good because when I was first diagnosed, I feel very well taken care of at this hospital. When I was first diagnosed I had a doctor who basically said, this is something you’re gonna have to live with. You know there are a couple of other treatments but you don’t want to do those and we’ll just put you on the ACE inhibitors you know. And I felt dismissed. I wanted to know more and I wanted to know what was possible.
Harms of risk score	Risk score could cause concern or anxiety • If there was a very high reading, what does that do to you mentally? • What about the situation where you log in and see the data and it’s like 99%, what is there for people like that with a mental reaction? Should ask patient if they want to know the risk score • it all depends on the patient. Some people might react in negative ways to that information or be overcome with anxiety. And so I think it’s important to ask the patient if they want to know • that’s a discussion between your physician and yourself, do you want to know? If something’s going wrong, I want to know. But it might not be the same with everybody else Physicians should check emotions on an ongoing basis • when you have the meeting and you get your diagnosis; there’s a lot to process. I processed my own emotion slowly and so the impact didn’t really hit until a few days later. But if I had known there’s a follow-up appointment in a week or two to go back with questions and ask my doctor, maybe and that would be a good time to say, how are you feeling? But in that diagnosis meeting, of course there might be an immediate reaction but it’s the long term reaction or the slower reaction that’s gonna affect you for a longer time • depending on the physician, patient relationship, getting over that hurdle of how should we approach the psychological impact of being diagnosed with something that we can’t do much for you other than renal replacement. The whole concept of renal replacement as a treatment option, it just sounds horrible. That doesn’t look like a really great treatment option. I don’t know if other people feel the same way. I hate the thought of begging for a kidney. I hate the thought of using any kind of dialysis. Dirt nap sounds a little better.
Desired adjunct information or format of risk score	On it’s own, risk score not useful • Narrowing it down to one number, one percentage seems like an oversimplification. It’s nice to have that, an idea of sort of where you sit statistically, but it doesn’t give you an idea of how you can change your lifestyle • You get a number, what does that mean? Is there a little report that comes with it? Discussion with physician essential • one caveat is if there was a little percentage chance that it could be a false negative. That this is something that could be addressed through doctor-patient counselling to reassure, just because it’s a low percent chance, you’re not out of the woods • this is supplementary rather than a replacement as part of the overall communications Clarifying purpose and timing • Is this a tool to find out if you have kidney problems? Not because you already had them and you’re monitoring? • So it’s not necessarily a tool that’s used after you have already been through a few years of it? How different variables influence risk score • Some questions about some of the metrics, it’s not clear if a change in proteinuria of 1.5 to 1.6 affects the potential number that I get scored at by a factor of 1 or 10 or 100. So the rating or the impact of the variations in values, is age increase by one year going to increase my risk by 10%, or does age increase by one year a very insignificant factor. So the ranking or the weighting of the individual metrics themselves would make it easier to better interpret the actual score • What is the scientific background, the interpretation of these numbers? What is the behind the scenes calculations? I would like to know a little bit more about how they came up with all this information, more than just a percentage • Some of the other metrics that I don’t think I have any control over are the MBST scores. Your biopsy results are created as a result of your test. I personally don’t ever want to have to go through that again. So I’ve repeating the biopsy to get a MEST score, no thank you; unless absolutely necessary and it’s a pretty high risk procedure anyway. So I don’t think I can change the outcome by asking for more biopsies, I actually make it worse. But the things I can do something about or changing my immunosuppressant medication levels, changing my diet, changing my coffee consumption or my alcohol consumption, whatever it takes Report score as a category • if it’s over a certain percentage, it’s flagged as very high rather than trying to grade between 50 and 99 or 100 • a translation table of what the different percentage ranges means to me directly. So a score of 0–5%, a score of 5–15%, a score of 15–25 indicates renal failure within one year, five years, ten years would be a better indicator for me than a number. So a correlation table of what this percentage could mean to me with respect to a need for renal replacement therapy. • I’m thinking of a green, yellow. If you’ve had lab work done. on a lab work result, you’ll get a green, yellow, red bar and it indicates you know if you’re in the yellow area that’s a sort of timeframe Report multiple scenarios based on differing variables • If my predication is 10-perecent with a five year forecast; is my prediction going to be 2% of the one year forecast? Is it linear or is it? So I’d like to know. I don’t want to know five years from now. I want to know 12 months from now is my decline immediate or is my decline longer term? • Can you play with numbers maybe? Have it like the loan thing where you’re trying to figure out your monthly payments, and you want a $500 amount payment. One year I get this, at two years I get this, at four years I get this. Report individual data in comparison with population • Some kind of a scale to know where you’re at with that number • Is there any way of knowing the rest of the population as a comparison? • knowing some statistics about people in my situation and my age with these scores. For a patient who looks like me, what is the likelihood that this treatment plan will be successful?
Other related desired information	Which factors influence disease even minimally that they could control • I had asked to see a dietician and then saw a naturopath as well, just to understand some other things that could help. If you know some of the things that we consume and some of the things that we shouldn’t be consuming. I don’t think what was communicated enough, I had to actually get the information • overall fitness I think was one of the suggestions about how I might be able to prolong but I don’t think I was given a whole lot of indicators of what can I do? What can I actually have an effect on? Is it nutrition? Is it weight? Is it exercise? • what else should we measure or what other factors that aren’t being taken into consideration with the tool today, could be that might be useful. So lifestyle choices I think is an indicator of prognosis if you have good or poor lifestyle choices and again, whether your obesity is a factor. It doesn’t appear to be anywhere in the metrics gathered. Is a patient who is extremely obese at a greater risk than somebody’s who weighs less? But if there’s an indicator that obesity is being taken into consideration, I think that might be something that at least would give patients an opportunity to say there’s something I can try and do something about. Even if it’s not a 100% on the weight scale [for the risk score], maybe it’s only a 1% or 2% impact, but it’s something the patient could use. So that would be another measure that I would think should be something to consider Informational/educational material • A huge part of it is education for the patient and actually trying to be able to understand what it is that they’re telling you. Because for some reason you start talking about creatinine and all these other values that you really don’t have a clue what they’re talking about and it’s very difficult to make any kind of decisions. Gauging the comprehension levels of the patient is very difficult for the physician in a 20-min consultation to actually determine if I understand what he’s telling me • when you first get your diagnosis you really don’t know anything about it. So the first thing you do, you try to look it up. And when you try to look it up, most of the websites are just like way over your head at first, right? Because you don’t understand what this means, what this number means or whatever, you’re just inundated. So if they had like a simplified, okay this is what this means like in laymen terms about all the aspects, really accessible … info, like on the website or something like that? It probably be a lot easier on someone

#### Need for risk score

Participants expressed two discrepant views about the value of a risk score. While they agreed that “more information is better”, they also questioned the need for the risk score when they already actively tracked their lab results through the hospital patient portal to monitor disease progression.

Did we need the risk calculator to get us on track and just have more self-awareness? No, I think we just need the right communication with our physician and the right information on how to interpret results from the lab

#### Potential benefit of risk score

Participants identified several benefits of the risk score: it could give them a sense of their health status and, given their prognosis, the score could influence life choices.

I certainly would make different life choices based on any percentage chance of having a significant risk of dialysis or even a transplant. It would accelerate travel choices, lifestyle choices, job choices and frankly would lead to quite a significant set of changes based on that information

Participants thought that, upon diagnosis, the risk score could function as a brief, simple summary of their condition that could then inform more detailed and ongoing patient-provider discussions to educate patients about the metrics that would be monitored over time to assess progression.

It is definitely overwhelming, the number of metrics you have to understand and learn about. It may be a good tool to just summarize because it is a really overwhelming and as soon as you get that diagnosis, it would be simple enough to have a number

Participants thought that the risk score was particularly important as a “conversation starter” for patients whose physicians might not be ideal communicators.

If it acts as a conversation starter, or someone asking questions, then that’s good. When I was first diagnosed I had a doctor who basically said, this is something you’re gonna have to live with. You know, there are a couple of other treatments, but you don’t want to do those and we’ll just put you on the ACE inhibitors. And I felt dismissed. I wanted to know more and I wanted to know what was possible.

Through repeated use, participants said the risk score could provide patients with information about the impact of treatment.

Knowing at the beginning and then repeating, to show we are making progress, or we are at least managing the condition would be a great indicator for me. It would show the efficacy of the treatment that I’m receiving.

They also emphasized that the health care professional providing information and education need not be a physician, and the risk calculator could equip non-physicians to assist with patient support.

I was very fortunate to have, like a team, the physician and the research scientist or technician, I’m not sure, help me interpret these things. So it wasn’t always a physician.

#### Potential harm of risk score

Participants also identified potential harm of the risk score, which could cause concern or anxiety. They noted that persons differ in their desire for information, and that physicians should first ask patients if they want risk data.

It all depends on the patient. Some people might react in negative ways to that information or be overcome with anxiety. And so I think it’s important to ask the patient if they want to know.

Participants said that, given surprise or shock upon first learning of their diagnosis, they experienced a delayed emotional reaction. Hence, they said physicians should schedule a follow-up meeting soon after diagnosis to address concerns or emotions, and that physicians should inquire about those feelings in subsequent periodic check-ups. This suggests that physicians should not rely on the risk calculator as the basis for a single, discrete conversation about prognosis.

#### Supplementary information

Participants felt that the risk score, a single number on its own, was not terribly useful, and offered several suggestions for supplemental information that should accompany the risk score to make it more useful for patients. However, they also said the risk score and accompanying information should be supplementary to, rather than a replacement for patient-provider discussion.

You get a number, what does that mean? Is there a little report that comes with it?

Participants wanted more information about how to interpret the risk score, and the weight of the variables that gave rise to the risk score. In large part, this was because they wanted to know which factors they could modify to improve their score, further emphasizing their desire for active involvement in their own health care and seeking to gain control.

It’s not clear if a change in proteinuria of 1.5 to 1.6 affects the potential number that I get scored at by a factor of 1 or 10 or 100. So the rating or the impact of the variations in values. Is age increase by one year going to increase my risk by 10%, or is age increase by one year a very insignificant factor. So the ranking or the weighting of the individual metrics would make it easier to better interpret the actual score.

Participants offered suggestions for alternative outputs to the single number risk score. They recommended a risk category along with a definition or explanation of the categories and associated health implications, particularly for higher risk scores. Participants also thought that a visual display would be more informative and appealing way to convey risk categories; for example, by using the green, yellow and red rubric.

So a score of 0 to 5%, a score of 5 to 15%, a score of 15 to 25% indicates renal failure within one year, five years, ten years would be a better indicator for me than a number. So a correlation table of what this percentage could mean to me with respect to a need for renal replacement therapy.

If it’s over a certain percentage, it’s flagged as very high rather than trying to grade between 50 and 99 or 100

They also agreed that seeing multiple scenarios based on different variables, for example, differing timeline, would be more useful than seeing only one risk score based on a single set of variables, likening this to mortgage amortization.

If my prediction is 10% with a five year forecast, is my prediction going to be 2% of the one year forecast? Is it linear? I don’t want to know five years from now. I want to know 12 months from now, is my decline immediate or is my decline longer term?

Another suggestion was to report individual risk data in comparison with similar patients as a way to gauge their own health status or progression.

Is there any way of knowing the rest of the population as a comparison? Knowing some statistics about people in my situation and my age with these scores. For a patient who looks like me, what is the likelihood that this treatment plan will be successful?

#### Other desired information

Apart from information to explain and supplement the risk score, participants also desired information on which lifestyle factors could prevent or reverse disease progression, even if they were not variables included in the risk score. They said that, even if the impact were small, they would want to do anything they could to improve their health status.

Is there anything at all that an individual can actually do to affect that number?

What other factors, that aren’t being taken into consideration with the tool today, might be useful. So lifestyle choices I think is an indicator of prognosis … It doesn’t appear to be anywhere in the metrics gathered … I think that might be something that at least would give patients an opportunity to say there’s something I can try and do something about. Even if it’s not a 100% on the weight scale [for the risk score], maybe it’s only a 1% or 2% impact, but it’s something the patient could use.

Participants also said there is a need for easy-to-understand information to read on their own, given that it was difficult to absorb the large amount of complex information conveyed to them during a clinical consultation.

A huge part of it is education for the patient and actually trying to be able to understand what it is that they’re telling you … Gauging the comprehension levels of the patient is very difficult for the physician in a 20-minute consultation

When you first get your diagnosis you really don’t know anything about it. So the first thing you do, you try to look it up. And when you try to look it up, most of the websites are way over your head at first, right? Because you don’t understand what this number means, you’re just inundated. So if they had a simplified, in layman terms, about all the aspects, really accessible, like on the website, it would probably be a lot easier

## Discussion

Using a focus group of IgAN patients who varied by age and disease severity, we identified both benefits and harms of implementing the IgAN RPT. Participants said physicians should first ask patients for permission to use the risk score, and that when used, it should be supplemental to, and not a replacement for patient-provider discussion. To make it more useful, they offered suggestions for information that should be provided along with the risk score to explain how the score was derived, how it will be used, and how to interpret the score. They also offered suggestions for alternative, possibly visual formats to more readily convey information about the risk score and its implications. Participants desired additional actionable information, potentially not included in the risk score, about what they could change or improve, even if impact on the risk score was small. These results will be used by the International IgAN Network to design and subsequently test a patient-oriented education tool specific to IgAN and that addresses the benefits and harms of the RPT that were identified in this study.

Prior research on RPTs examined the perspective of physicians pertaining to diabetes, cardiovascular disease, post-operative nausea, and physical therapy [13–16, 19–21]. While some of those studies identified potential patient barriers (i.e. understanding results), they were identified by physicians and not by patients. Hence, this study is unique in that it directly elicited views about RPTs from patients, and for a condition such as IgAN that has not been previously studied. Furthermore, by using a qualitative approach, we gathered rich, detailed information about a wide variety of factors that may influence whether and how physicians employ RPTs in IgAN. Overall, this suggests that if RPTs are not designed to meet patient needs, they may be rejected by patients, or contribute to concern and anxiety among patients. This finding potentially explains prior research showing that RPTs were not used in practice [13–16], and confirms prior research showing that condition-specific communication tools were more likely to be adopted and offered greater benefit when aimed at both patients and clinicians [27].

This study provides useful insight on how to enhance the implementation of RPTs in IgAN by designing them so that they are easy to understand and apply by patients. In this study, patients said that the single risk score would be more useful if visually displayed and accompanied by information on how to interpret the risk score, risk categories, health implications, and modifiable risk factors, with the provision of multiple scenarios that vary by variables such as timeline, and with comparison to similar patients. Although the results of this study will be used to design a patient-oriented education tool specific to IgAN, it is not known if these preferences also apply to RPTs for patients with other conditions. As such, those who create RPTs should employ a user-centred design approach, which engages end-users in all development stages including design, pilot-testing and trialing [23]. Patients in this study emphasized that physicians should not rely on the RPT as the basis for a single, discrete conversation and should instead use the RPT to inform additional, ongoing communication. This pertained to the emotional reaction to a diagnosis of IgAN, which patients described as delayed. This finding underscores that physicians must address the multidimensional nature of PCC, conceptualized as 28 elements organized in six domains: foster patient-clinician relationship, exchange information, recognize and respond to patient emotions, manage uncertainty, make shared decisions, and enable patient self-management [29, 30]. Patients desired more information and education on lifestyle factors they could modify, thereby taking an active role in their illness, and assuming control for at least some part of it. A meta-review of self-management for individuals with chronic kidney disease revealed that patients perceived a shallow relationship with health care providers and desired greater educational and emotional support so that they could optimize self-management and quality of life [31].

Patients in the current study also highlighted that other members of the multidisciplinary team could assist physicians in providing information and education. Multidisciplinary care for chronic kidney disease often involves nephrologists, nurses, dieticians, social workers, and pharmacists, and has been associated with improved health-related quality of life and patient self-care abilities [32, 33]. Still, research has identified numerous physician-specific barriers of RPT use, which in turn, may limit use by other members of the multidisciplinary team or by patients. Therefore, in addition to studying patient uptake of RPTs, ongoing research must in parallel investigate how to optimize the design and implementation of RPTs from the physician perspective. Given divergent physician barriers (e.g. views about strength or validity of underlying evidence, logistics of incorporating RPTs into practice), multiple strategies may be needed to promote physician adoption of RPTs. Of note, the Kidney Disease Improving Global Outcomes recent 2020 update to the international guidelines for the management of patient with IgAN now specifically recommend that physicians use the RPT to risk stratify all patients with IgAN (https://kdigo.org/guidelines/gn/). We anticipate that this guideline will encourage and facilitate physician use of the RPT in clinical practice.

Similar to prior research, our results show that patients desire and benefit from supplementary information and visual aids in additional to risk statistics [34, 35]. However, much of that prior research did not elicit views directly from patients, or about the design of an RPT or accompanying information. Such examples include a review of issues pertaining to communicating numerical data in general, and not specifically risk data [34]; consensus generated by a panel of 14 researchers on how to communicate risk [35]; and interviews with 15 patients about the acceptability and use of an RPT for colorectal cancer screening in which their expressed views pertained to value for the RPT if it steered them to the fecal immunochemical test, which they preferred over colonoscopy [36]. Tang et al. interviewed 15 stroke patients about an RPT for post-stroke dementia, in which patients identified several benefits (timely diagnosis, time to prepare, reassurance) and challenges (anxiety about a dementia diagnosis) but did not describe how to design or implement the RPT [37]. Thus, our study is unique in that we employed a qualitative approach to thoroughly understand how to optimize the design and implementation of an RPT, in our case, specifically for IgAN. Still, future research is warranted to synthesize published research similar to Tang et al. [37] and our study on patient views or experiences with RPTs, information of use to those developing and/or implementing RPTs or accompany patient information or education.

Several themes also suggest that, when implementing the RPT in IgAN, physicians or other members of the multidisciplinary team should provide patients with brief education about the purpose of the RPT. For example, patients initially questioned why an RPT was needed if they themselves could follow lab test results via online patient portals, signaling confusion about short-term changes in lab tests versus the long-term scenario offered by the RPT. Patients also perceived that they could repeat the RPT after changes in diet or lifestyle; hence, brief education may be needed to explain that RPT data is based on similar patients, and it may not reflect individual reduction in risk based on lifestyle changes.

This study featured both strengths and limitations. We employed rigorous qualitative methods that complied with reporting standards [24–28]. In so doing, we generated thorough insight on how to enhance the design of an RPT for IgAN patients, which may lead to improved implementation in practice, and associated benefits for patients. Although IgAN is a common type of auto-immune kidney disease, it is still rare with an incidence rate 30-fold less than more usual non-immune types of kidney disease such as diabetes and hypertension [7]. This likely contributed to the small sample size of our study. While the participants varied in age and represented a spectrum of disease severity (e.g. eGFR, proteinuria), other patient characteristics that could influence views were uniform (i.e. all Caucasian and male). Also, given the volunteer nature of this research, the participants may have represented those who are proactive about seeking and using health information. As we conducted a single focus group, thematic saturation could not be assessed with a different group of participants. However, this study comprised the first step in a widely-used process for developing and evaluating interventions, concept generation, typically involving brief consultation with a small group of end-users to gather preliminary feedback that is used to refine the intervention prior to engaging end-users in its further testing and evaluation [22, 23]. Furthermore, the key to sample size in qualitative research is “information power”: when the study goal is narrow, the questions few and specific, and the dialogue rich, as was the case in this study, the fewer participants are needed [38]. Despite these limitations, the insights generated several implications for policy and practice, which raise some issues that warrant ongoing research including: explore views among patients with diverse characteristics, assess views about RPT use among nephrologists, and develop and implement the IgAN RPT with the features suggested by patients and evaluate its impact on a range out outcomes such as patient-provider communication, satisfaction with the care experience, confidence to self-manage, and quality of life. Future development and evaluation of patient-oriented information or education pertaining to an IgAN RPT should engage patients and family with diverse characteristics across multiple countries including China, Japan, and other countries in South and East Asia where IgAN is a common cause of kidney disease.

## Conclusions

Patients with IgAN who participated in a single qualitative focus group identified numerous preferences for the design of an IgAN RPT (visual display, information on how to interpret the risk score, risk categories, health implications, modifiable risk factors, multiple scenarios, and comparison with similar patients). They also provided important suggestions on how it should be implemented in practice (physicians should ask permission to use it, it should not replace patient-provider discussion, it should be accompanied by self-management education so that patients can take an active role, patients appreciate information from members of the multidisciplinary team in addition to physicians, and physicians should monitor emotions or concerns on an ongoing basis). We will use this knowledge to refine the design of a patient education tool to support use of the IgAN RPT. We suggest this approach and the findings can be used to design RPTs for patients with kidney conditions other than IgAN and with other organ progressive disease conditions.

## Supplementary information

**Additional file 1.** Focus group guide. Lists questions used and time allotted for each questions during the focus group.

**Additional file 2.** Consolidated Criteria for Reporting Qualitative Research

## Data Availability

All data generated or analysed during this study are included in this published article [and its supplementary information files].

## Abbreviations

IgAN: IgA nepropathy
PCC: Patient-centred care
RPT: Risk prediction tool
