# Supplementary material for: How to optimize the design and implementation of risk prediction tools: focus group with patients with IgA nephropathy
Source: BMC Med Inform Decis Mak. 2020 Sep 16;20:231. doi: 10.1186/s12911-020-01253-4 (PMC7493917; doi:10.1186/s12911-020-01253-4)

**Additional File 1. Focus group guide**

**Opening Remarks + General Questions (10 min)**

- As people with lived experience of nephropathy, we would like your opinions on the use of an IGA nephropathy risk calculator
- A risk calculator is a paper or electronic tool that considers patient characteristics and generates a number that can be used by the doctor to determine if there is a chance that a patient’s kidney function will worsen and how to best care for them – this can help patients and physicians together decide on the best treatment
- First, I will show you what the risk calculator looks like, and then ask a few questions about it; the nephrologist will use your ideas to improve the design of the risk calculator

**Demonstration with power point (5 min)**

**Focus group questions (45 min)**

| Question | Prompts | Time (min) |
| --- | --- | --- |
| 1. How would you use information about whether you do or do not have a future risk of worsening kidney function? | Is that information of interest to you? Why or why not?  How would that information impact you or your life?  *Getting worse includes a decrease in kidney function by 50% or more, or a total kidney failure needing dialysis and/or a kidney transplant* | 10 |
| 1. How could the risk calculator help you discuss your health with your doctor? | Would it help your doctor communicate results?  Would it help you: Understand results? Know what to ask? Understand whether or not you would benefit from treatment? | 10 |
| 1. How could the format and output of the risk calculator be improved so that it is more useful? | Format: Prompt for web-based, app for phone, program for desk-top computer or computer network, or communicated to you by your physician (on paper, verbally)  Output: Numerical risk score, overall risk, measure of uncertainty (i.e. confidence interval), risk category (i.e. low, medium, high), numeric vs graphic or both | 10 |
| 1. In addition to the risk calculator, is there anything else that would help patients discuss the risk calculator results with their nephrologist | Facilitators: Training on use of the calculator, information for patients to support informed decision-making, shared decision-making tools to support communication between patients and physicians, information about what risk means, other information from your physician to help you understand your risk  Barriers:  Not interested in that level of detail, concern about understanding the information and its implications | 10 |
| 1. Conclusion | Any final suggestions or questions | 5 |

--- Thank you for taking the time to speak with me ---


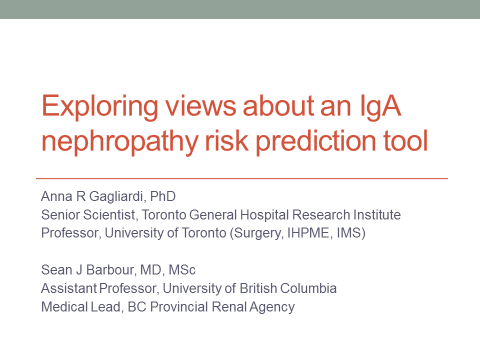


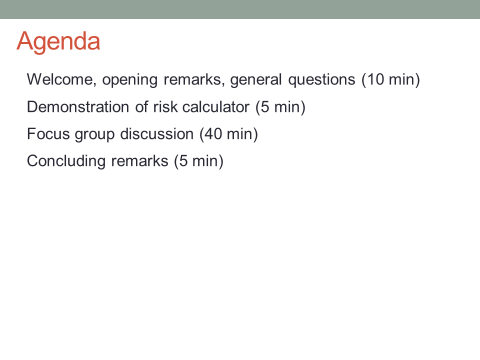


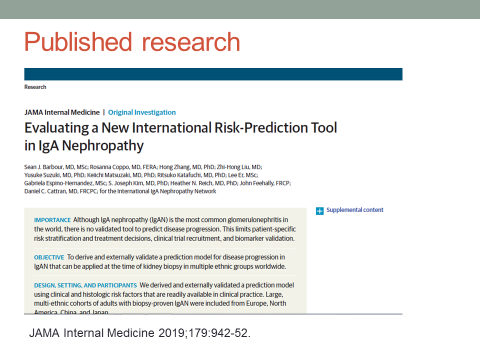


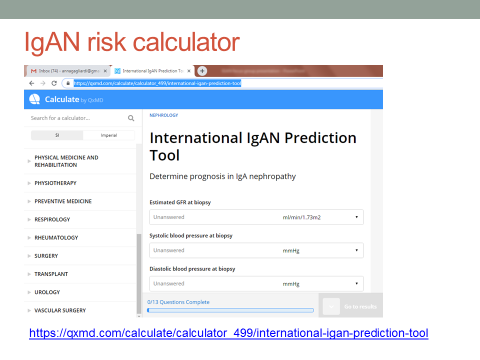


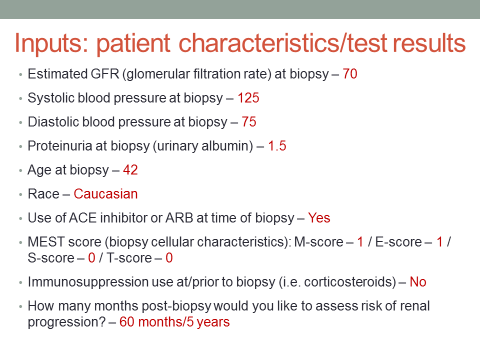


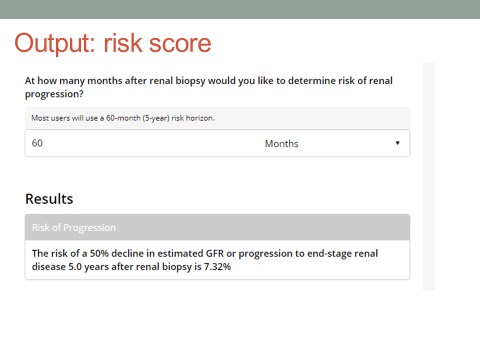

Supplement: Supplementary file 1 — Additional file 1. Focus group guide. Lists questions used and time allotted for each questions during the focus group. [file 12911_2020_1253_MOESM1_ESM.docx]
